# Nanoparticle-Mediated Physical Exfoliation of Aqueous-Phase Graphene for Fabrication of Three-Dimensionally Structured Hybrid Electrodes

**DOI:** 10.1038/srep19761

**Published:** 2016-01-27

**Authors:** Younghee Lee, Hojin Choi, Min-Sik Kim, Seonmyeong Noh, Ki-Jin Ahn, Kyungun Im, Oh Seok Kwon, Hyeonseok Yoon

**Affiliations:** 1 Alan G. MacDiarmid Energy Research Institute, School of Polymer Science and Engineering, Chonnam National University, 77 Yongbong-ro, Buk-gu, Gwangju 61186, South Korea; 2 Department of Polymer Engineering, Graduate School, Chonnam National University, 77 Yongbong-ro, Buk-gu, Gwangju 61186, South Korea; 3 BioNanotechnology Research Center, Korea Research Institute of Bioscience and Biotechnology, 125 Gwahak-ro, Yuseong-gu, Daejon 34141, South Korea

## Abstract

Monodispersed polypyrrole (PPy) nanospheres were physically incorporated as guest species into stacked graphene layers without significant property degradation, thereby facilitating the formation of unique three-dimensional hybrid nanoarchitecture. The electrochemical properties of the graphene/particulate PPy (GPPy) nanohybrids were dependent on the sizes and contents of the PPy nanospheres. The nanohybrids exhibited optimum electrochemical performance in terms of redox activity, charge-transfer resistance, and specific capacitance at an 8:1 PPy/graphite (graphene precursor) weight ratio. The packing density of the alternately stacked nanohybrid structure varied with the nanosphere content, indicating the potential for high volumetric capacitance. The nanohybrids also exhibited good long-term cycling stability because of a structural synergy effect. Finally, fabricated nanohybrid-based flexible all–solid state capacitor cells exhibited good electrochemical performance in an acidic electrolyte with a maximum energy density of 8.4 Wh kg^−1^ or 1.9 Wh L^−1^ at a maximum power density of 3.2 kW kg^−1^ or 0.7 kW L^−1^; these performances were based on the mass or packing density of the electrode materials.

Nanostructured hierarchical hybrid materials, which have superior interfacial contacts and volume utilization to the conventional materials used in electrical devices, have enabled improved device performance in the field of energy storage and conversion^1^,^2^,^3^. As a result of the enhanced energy density, high power, and cycle stability of these materials, nanohybrid-based electrochemical capacitors, in particular, have received considerable attention as promising energy-storage devices^4^,^5^,^6^,^7^. The ability of an electrochemical capacitor to store electrical energy is highly dependent on the specific surface area, surface functionality, porosity of the electrode material, and electrical properties. Graphene, which has a unique two-dimensional atomic-scale planar structure, exhibits outstanding mechanical properties and electrical and thermal conductivity. Thus, considerable research on the development of graphene-based electrode materials has been conducted^8^,^9^,^10^,^11^. As a representative example, reduced graphene oxide has been widely studied despite its tedious multi-stage synthetic process and low cost-effectiveness. However, this material suffers from defects caused by irreversible oxidation/reduction reactions, which prohibit its widespread practical application.

In addition, the use of conducting polymers in a variety of applications (including electrochemical capacitors) has been extensively explored^12^,^13^,^14^,^15^,^16^,^17^; this is because of the ease of synthesis and functionalization, reversible redox properties, and flexibility of these materials. In order to further improve their properties, considerable effort has been devoted to tailoring the sizes and morphologies of the nanoscale components of conducting polymers^18^,^19^,^20^,^21^. However, the majority of polymers, including conducting polymers, are inherently unstable at this scale; this has impeded research on polymer nanomaterials.

Finally, pseudocapacitors have been shown to exhibit enhanced energy density with higher power than electric double-layer capacitors. However, pseudocapacitor performance is also limited because of the low cycle stability of the electrode materials. Therefore, recent research trends have focused on the rational combination of electric double-layer capacitive and pseudocapacitive electrode materials to overcome these problems^22^,^23^,^24^,^25^,^26^,^27^,^28^.

Herein, we report a simple, physical strategy for fabricating three-dimensionally structured graphene/particulate polypyrrole (GPPy) nanohybrids and an evaluation of their electrochemical properties. Using this approach, monodispersed polypyrrole (PPy) nanospheres can be effectively intercalated between graphene layers without defect formation, leading to alternately stacked GPPy nanohybrids. These nanohybrids have both electric double-layer capacitive and pseudocapacitive components, and, importantly, their morphologies and microstructures are tunable via adjustment of the intercalated PPy nanosphere content to regulate their electrical/electrochemical properties.

## Results and Discussion

**Figure 1:**
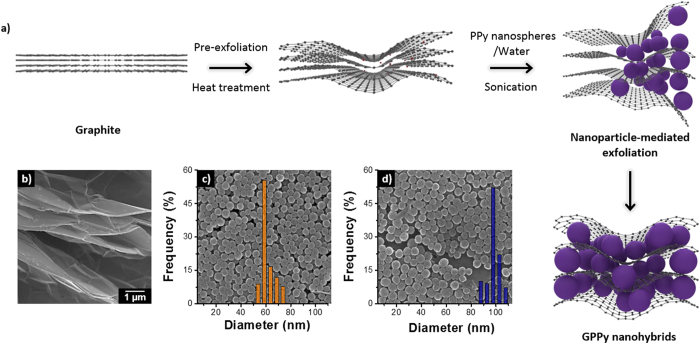
(**a**) Schematic illustration of GPPy nanohybrid fabrication. SEM images of the (**b**) graphite precursor, (**c**) 60 nm–diameter PPy nanospheres, and (**d**) 100 nm–diameter PPy nanospheres.

**Figure 2:**
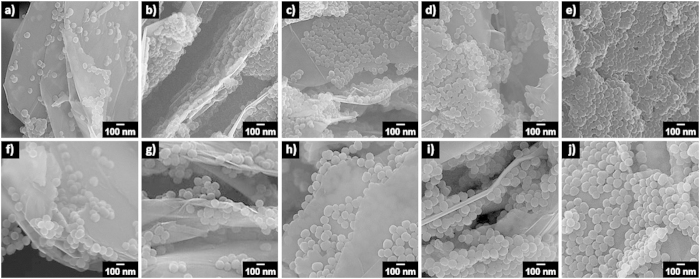
SEM images of the GPPy nanohybrids (*m*GPPy*n*) with different PPy/graphite weight ratios (*m*) and PPy nanosphere diameters (*n*). (**a**) 2GPPy60, (**b**) 4GPPy60, (**c**) 6GPPy60, (**d**) 8GPPy60, (**e**) 10GPPy60, (**f**) 2GPPy100, (**g**) 4GPPy100, (**h**) 6GPPy100, (**i**) 8GPPy100, and (**j**) 10GPPy100.

GPPy nanohybrids were fabricated using a simple physical synthesis route, as illustrated in Fig. 1a and described above. For efficient intercalation of the PPy nanospheres, the parent graphite was pre-treated using small molecules, and the resultant graphite was sonicated with the nanospheres in an aqueous solution. Concentrated dispersions were readily obtained through mild sonication, which was used to prevent degradation of the material properties. Hydrophobic interactions between the nanospheres and graphene in the aqueous solution facilitated intercalation of the nanospheres between the graphene layers. Figure 1b shows a scanning electron microscopy (SEM) image of the graphite precursor. It is apparent from Fig. 1c,d, which show SEM images of the PPy nanospheres, that the nanospheres with diameters of approximately 60 (61 ± 5.4 nm) and 100 nm (98 ± 5.0 nm), respectively, had reasonably narrow size distributions. The nanospheres were introduced into the graphene layers at several different PPy/graphite weight ratios. The actual amount of nanosphere loaded into the nanohybrid increased logarithmically with increasing weight ratio (see Table S2 in Supplementary Information). Figure 2 shows typical SEM images of the GPPy nanohybrids with different PPy nanosphere contents. Interestingly, the nanospheres adhered well to both surfaces of the exfoliated graphene, creating a unique three-dimensional architecture with nanoscale-sized features. Compared to the 100 nm–diameter nanospheres, the 60 nm–diameter nanospheres had a higher surface coverage because of the higher surface-to-volume ratio. In the case of the 60 nm nanospheres, the surface of the graphene was almost covered by the nanospheres at a 10:1 PPy/graphite weight ratio. The majority of the exfoliated graphene sheets had features a few micrometers in size, indicating that nanoparticle intercalation proceeded effectively without serious destruction of the carbon basal planes.

**Figure 3:**
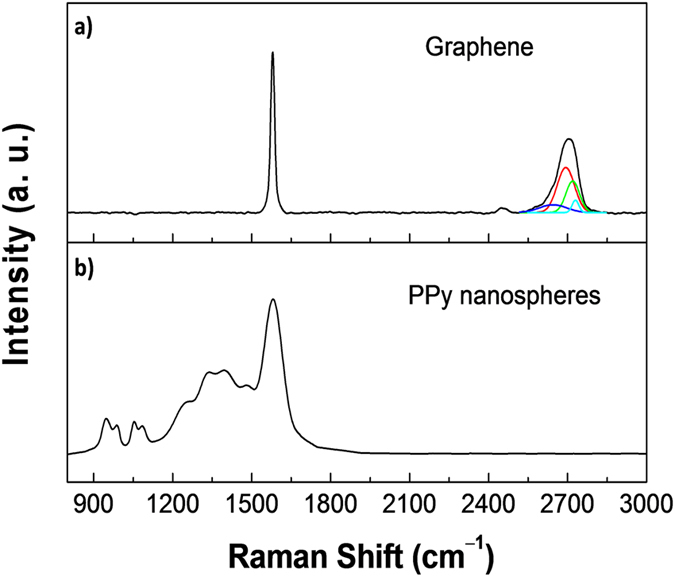
Representative Raman spectra collected from the GPPy nanohybrids (excitation wavelength, λ_exc_ = 532.13 nm). (**a**) Graphene (the deconvoluted components of the 2D peak are given) and (**b**) PPy nanospheres.

**Figure 4:**
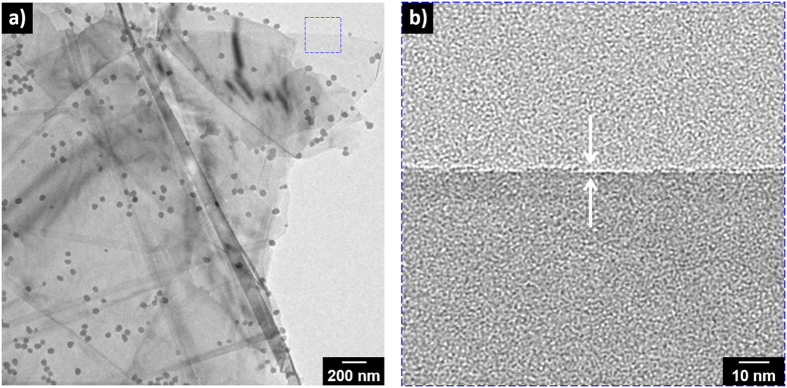
Representative TEM images of the GPPy nanohybrids (4GPPy60). The selected region (blue square) in image (**a**) was magnified and presented in (**b**).

Raman spectroscopy was used to qualitatively characterize the GPPy nanohybrids. Figures 3a,b display *in situ* Raman spectra of graphene and PPy, respectively, obtained from the 8GPPy100 sample. A number of points should be noted: First, there is no D peak in the graphene Raman spectrum, which indicates that the nanospheres efficiently exfoliated the graphene layers from the graphite. Deconvolution of the 2D peak revealed four components, suggesting that the exfoliated graphene was as thin as a bilayer^29^,^30^. In fact, electron microscopy allowed observation of the exfoliated single-layer graphene, as seen in Fig. 4. However, it was difficult to obtain a characteristic Raman spectrum of the single-layer graphene from the nanohybrid. Secondly, the characteristic peaks of the PPy were observed at 944, 986, 1053, 1086, 1256, 1336/1395, 1481, and 1581 cm^−1^; these were attributed to dication ring deformation, radical cation ring deformation, radical cation symmetrical C–H in-plane bending, dication symmetrical C–H in-plane bending, antisymmetrical C–H in-plane bending, antisymmetrical C–N stretching, reduced C=C stretching, and oxidized C=C stretching, respectively^26^,^31^,^32^. In particular, the oxidized C=C backbone stretching peak (the intensity ratio of the 1581 cm^−1^ peak to 1481 cm^−1^ peak was 2.2) indicates that the PPy nanospheres retained the oxidation level and were, thus, in a conductive state.

**Figure 5:**
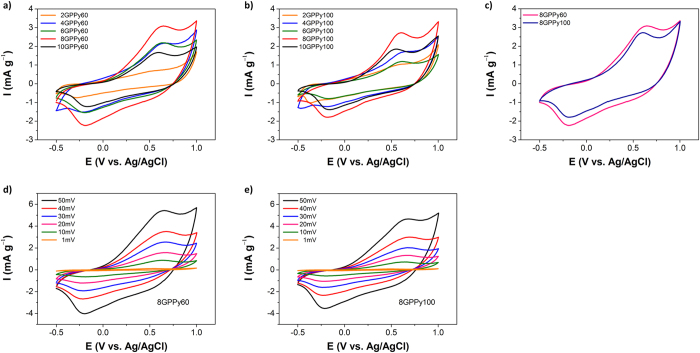
Electrochemical properties of the GPPy nanohybrids measured in 1 M sulfuric acid. CV curves of (a) GPPy60 and (**b**) GPPy100 samples with different PPy/graphite weight ratios at a 25 mV s^−1^ scan rate; (**c**) CV curves of 8GPPy60 versus 8GPPy100 recorded at a 50 mV s^−1^ scan rate; and CV curves of (**d**) 8GPPy60 and (**e**) 8GPPy100 recorded at scan rates ranging from 1–50 mV s^−1^.

To evaluate the potential of GPPy nanohybrids as electrode materials for electrochemical capacitors, the electrochemical properties of the fabricated samples were first investigated using cyclic voltammetry (CV) in a three-electrode cell (Fig. 5). The CV curves were recorded at a scan rate of 25 mV s^−1^ in the −0.5–+1 V potential range using a 1 M H_2_SO_4_ solution. All GPPy nanohybrids exhibited a pair of redox peaks that were centered at approximately 0.6 and −0.2 V, the intensities of which depended on the PPy nanosphere content. In other words, the redox activity of the PPy nanospheres was well-preserved in the nanohybrids. Interestingly, the integrated areas of the GPPy60 and GPPy100 nanohybrid CV curves (Fig. 5a,b, respectively), which represent the capacitance, increased with increasing PPy/graphite weight ratio from 2:1 to 8:1 and then decreased for the samples with a 10:1 PPy/graphite weight ratio. Based on both SEM observations and CV analysis, an optimum degree of surface coverage of the graphene by the nanospheres, which significantly affects the nanohybrid electrochemical performance, should exist. The integrated areas of the CV curves of the nanohybrids with the same PPy nanosphere content were also dependent on the nanosphere size. For comparison, CV curves of the 8GPPy60 and 8GPPy100 samples were recorded at a scan rate of 25 mV s^−1^, and the results are presented in Fig. 5c. Compared to the 100 nm–diameter nanospheres, the 60 nm–diameter nanospheres covered more of the graphene surface, which may account for the enlarged CV curve. Figures 5d,e display representative CV curves measured at scan rates ranging from 1–50 mV s^−1^. The integrated areas of the CV curves increased proportionally with increasing scan rate, supporting the conclusion that the GPPy nanohybrids have good electrochemical activity and kinetic reversibility.

**Figure 6:**
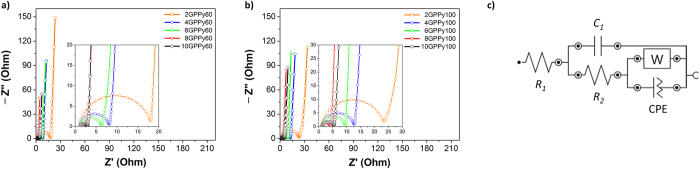
EIS Nyquist plots of the GPPy nanohybrids measured in 1 M sulfuric acid. (**a**) GPPy60 and (**b**) GPPy100 samples in the 100 mHz to 1 MHz frequency range. (**c**) An equivalent circuit model.

To further elucidate the electrochemical properties of the samples, the GPPy nanohybrids were further characterized using electrochemical impedance spectroscopy (EIS) with the same setup as that used for the CV tests (Fig. 6). The GPPy nanohybrids produced Nyquist plots that comprised an arc at high frequencies and a diagonal line with a positive slope at low frequencies (Fig. 6a,b, respectively). The real impedance component (Z′) corresponding to the peak of the arc, which is indicative of the charge-transfer resistance (*R*_ct_), was dependent on the PPy nanosphere content of the nanohybrid samples. Before exfoliation, the graphite was calculated to have a high *R*_ct_ of 31 Ω and low capacitance of 5.6 μF (see Figure S4 and Table S1 in Supplementary Information). In contrast, in both the GPPy60 and GPPy100 samples, the *R*_ct_ value decreased gradually as the PPy/graphite weight ratio increased from 2:1 to 8:1 and then increased slightly at the 10:1 PPy/graphite weight ratio; this implies that electron transfer at the electrolyte/GPPy nanohybrid electrode interface is more effective than that at the electrode consisting of pre-exfoliated graphite. The rise in *R*_ct_ at the 10:1 weight ratio is likely due to excess surface coverage by the nanospheres or increased inter-nanosphere contact resistance in the nanohybrid. In terms of the nanosphere sizes, the arcs of the Nyquist plots of GPPy60 had smaller diameters than those of the GPPy100 samples. These trends are commensurate with the results obtained from the CV analyses, implying that the effective surface areas and redox activities of the nanohybrids determine the *R*_ct_ value. The EIS data was further inspected through fitting to an equivalent circuit model. Figure 6c illustrates the best-fit circuit model, which is composed of two resistors (*R*_1_ and *R*_2_), a capacitor (*C*_1_), a Warburg element (W), and a constant phase element (CPE) (see Table S1 in Supplementary Information for the calculated values of the parameters). The *R*_1_ and *R*_2_ values correspond to the electrolyte resistance and *R*_ct_, respectively, while the *C*_1_ and CPE values are derived from the pseudocapacitance of PPy and electric double-layer capacitance of graphene, respectively^33^,^34^. The calculations indicated that both the *C*_1_ and CPE values were also dependent on the PPy nanosphere content, with similar tendencies as those revealed by the CV analyses.

**Figure 7:**
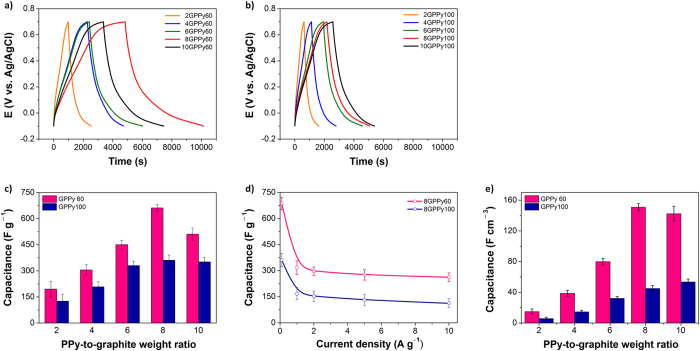
Capacitive performances of the GPPy nanohybrids measured in 1 M sulfuric acid with a three-electrode configuration. Galvanostatic charge/discharge curves of (a) GPPy60 and (**b**) GPPy100 (only the discharge curves of 8GPPy100 and 10GPPy100 were enlarged for comparison and are provided in Supplementary Information, Figure S3) with different PPy/graphite weight ratios at a 0.1 A g^−1^ current density. Specific capacitances of GPPy nanohybrid electrodes with (**c**) different PPy/graphite weight ratios at a 0.1 A g^−1^ current density and (**d**) an 8:1 PPy/graphite weight ratio versus the current density. (**e**) Volumetric capacitances of the GPPy nanohybrids calculated from (**c**).

The specific capacitances of the GPPy nanohybrids were calculated directly using galvanostatic charge/discharge measurements (Fig. 7). A potential window of 0.8 was chosen after taking the CV data into account, and the current density was set at 0.1 A g^−1^ to facilitate a direct performance comparison between the individual samples. All charge/discharge curves exhibited good reversibility during the charge/discharge process within the corresponding potential window, and the *IR* drop during the initial discharge was almost negligible. The slopes of the curves were non-linear because of the PPy-mediated faradaic reactions. As can be seen in Fig. 7a,b, the discharge times of the GPPy60 and GPPy100 samples increased within the same potential window for nanohybrids with increasing PPy nanosphere content up to a PPy/graphite weight ratio of 8:1 and then decreased at the 10:1 weight ratio; this was likely due to overloading of the PPy nanospheres, which have relatively low specific capacitances (i.e., 253 and 184 F g^−1^ at 0.1 A g^−1^ for the 60 and 100 nm–diameter PPy nanospheres, respectively). The discharge time was proportional to the discharge capacitance. Figure 7c summarizes the specific capacitance values calculated from the discharge curves. The maximum specific capacitance of the GPPy60 samples was 662 F g^−1^, which was obtained at an 8:1 PPy/graphite weight ratio; this result is significantly higher than that of pre-exfoliated graphite alone (<1 F g^−1^) and approximately 2.5 times higher than that of PPy nanospheres alone (253 F g^−1^). These results strongly support the conclusion that the combination of PPy nanospheres and graphene layers induces a synergistic effect that enhances the capacitive performance. This effect occurs because the inter-nanosphere pores between the graphene layers facilitate ion transport, enabling the pseudocapacitance of the PPy nanospheres to be effectively delivered to the current collector without significant inter-nanosphere contact resistance through the aid of the graphene layer. The GPPy60 samples had a greater increase in the specific capacitance than the GPPy100 samples over the entire weight-ratio range, confirming that the specific capacitance of the nanohybrids was determined by both the PPy nanosphere size and loading amount. The charge/discharge behavior at current densities ranging from 5 × 10^−2^–10 A g^−1^ was also explored: In general, the specific capacitance decreased at higher current densities, which is mainly because of the limited ion mobility in the electrolyte. At a PPy/graphite weight ratio of 8:1, the specific capacitance of the GPPy60 samples was higher than that of the GPPy100 samples for all current densities (Fig. 7d).

One of the major bottlenecks for nanostructured electrode materials in electrochemical capacitors is low volumetric capacitance^35^,^36^. The packing density of the nanohybrid increased with increasing nanosphere content. Under several assumptions (see Supporting Information), the theoretical packing densities of the GPPy nanohybrids with different nanosphere contents were calculated, and the gravimetric capacitance (F g^−1^) results shown in Fig. 7c were converted to volumetric capacitance (F cm^−3^). As can be seen in Fig. 7e, the gravimetric capacitance trend was almost duplicated by the volumetric capacitance, indicating that our synthetic approach is advantageous for the fabrication of electrode materials with enhanced gravimetric as well as volumetric capacitance.

**Figure 8:**
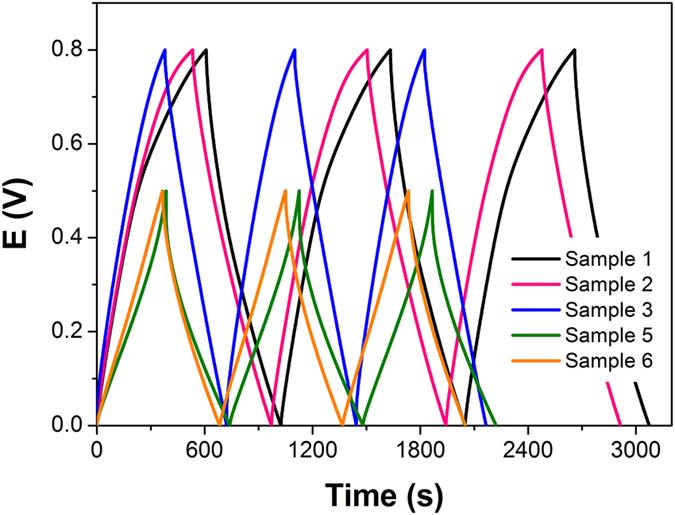
Typical galvanostatic charge/discharge curves of 8GPPy60 capacitor cells with different electrolytes, separators, and current collectors recorded at a 0.1 A g^−1^ current density.

The GPPy nanohybrid with the best performance, i.e., 8GPPy60, was further tested in a two-electrode system. The 8GPPy60 was assembled into a symmetric electrode capacitor cell with several different electrolyte, separator, and current collector combinations. Figure 8 presents typical galvanostatic charge/discharge curves of the 8GPPy60 capacitor cells, and Table 1 summarizes the main performance characteristics of the cells. It was found that the capacitive performance of the 8GPPy60 capacitor cells was dependent on the electrolyte and separator type. Specifically, the voltage range of the cell with the basic electrolyte (6 M KOH) was narrower than those of the cells with the acidic (1 M H_2_SO_4_) and neutral (1 M Na_2_SO_4_) electrolytes. Compared to the neutral electrolyte, the acidic and basic electrolytes facilitated higher specific capacitance in the cell. Apart from the cell with the neutral electrolyte, the separator appeared to have little effect on the cell performance. Considering the voltage range and specific capacitance, the system comprising an acidic electrolyte with cellulose separator was the optimal setup for the GPPy nanohybrids.

**Figure 9:**
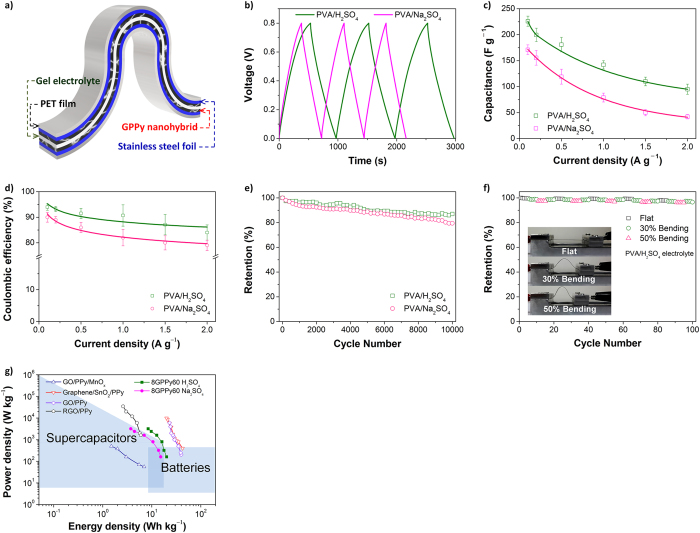
Flexible, all–solid state 8GPPy60 electrochemical capacitor cells. (**a**) Schematic of the all–solid state cell structure; (**b**) representative galvanostatic charge/discharge curves at a 0.1 A g^−1^ current density for different electrolytes; (**c**) specific capacitances at different current densities; (**d**) coulombic efficiency at different current densities; (**e**) long-term cycling stability of the cells with different electrolytes; (**f**) capacitance variation for different bend radii (insets show the consequent changes in the cell shape at each bend radius); and (**g**) Ragone plots of the all–solid state cells and other previously reported carbon/conducting-polymer materials (liquid electrolyte–type cells).

Lastly, a flexible all–solid state electrochemical capacitor cell was fabricated using 8GPPy60 as the electrode material. Figure 9a shows the structure of the all–solid state cell, in which an electrolyte-containing polyvinyl alcohol (PVA) gel and two stainless-steel foils were used as the separator and current collectors, respectively. As the electrolyte, 1 M H_2_SO_4_ or 1 M Na_2_SO_4_ solutions were employed. Figure 9b shows the galvanostatic charge/discharge curves of the cells, which had a PVA/H_2_SO_4_ or PVA/Na_2_SO_4_ gel separator. The discharge capacitance of the cell had a maximum value of 225 F g^−1^ when the PVA/H_2_SO_4_ gel separator was used. Sulfuric acid has a smaller cation than sodium sulfate and can serve as a doping agent during the charge/discharge process^37^. Therefore, as expected, the use of the PVA/H_2_SO_4_ gel separator led to a 4–9% increase in the specific capacitance over that of the PVA/Na_2_SO_4_-containing cell (Fig. 9c). Similarly, the coulombic efficiency of the cell containing the PVA/H_2_SO_4_ gel separator was better than that of the PVA/Na_2_SO_4_-containing cell (Fig. 9d). The specific capacitances of the cells were monitored over 10 000 cycles to examine their cycling stability. The all–solid state cell exhibited ~90% retention of the initial capacitance at 5000 cycles and more than 80% retention at 10 000 cycles (Fig. 9e). Considering the poor long-term cycling stability of pseudocapacitive electrodes, it is evident that the unique structure of the GPPy nanohybrids effectively prevents the electrical and electrochemical degradation of PPy.

To evaluate the flexibility of the capacitor cell, the specific capacitance was examined under bending conditions (Fig. 9f). An approximately 2% minor fluctuation in capacitance was observed during the bending cycle (0–50%), demonstrating the excellent flexibility of the cell. Figure 9g shows the Ragone plot of the flexible, all–solid state 8GPPy60 capacitor cell, depending on current density and in comparison with other previously reported carbon/conducting-polymer liquid electrolyte capacitors^38^,^39^,^40^,^41^. Compared with the other liquid-electrolyte capacitors, the all–solid state 8GPPy60 capacitor cells exhibited similar or even better performance. Notably, the 8GPPy60 cells demonstrated the ability to deliver an energy density of 8.4 Wh kg^−1^ (1.9 Wh L^−1^) at a high power density of 3.2 kW kg^−1^ (0.7 kW L^−1^) with a PVA/H_2_SO_4_ gel separator.

## Conclusions

The effective intercalation of PPy nanospheres into graphene layers created a unique three-dimensional hybridized nanoarchitecture. The GPPy nanohybrids were found to have excellent capacitive performance, which was the result of both structural and qualitative synergistic effects. Further, the performance was found to be strongly dependent on the PPy nanosphere size and loading amount. The long-term cycling stability of the nanohybrid-based capacitor cells was also good, which was attributed to the improved electrochemical stability of the PPy in the nanohybrid structure. Ultimately, nanohybrid-based all–solid state capacitor cells were successfully fabricated and exhibited good energy and power densities along with good mechanical flexibility. There is huge demand for graphene exfoliation^42^,^43^,^44^,^45^,^46^,^47^. It is believed that our nanosphere-mediated graphene exfoliation technique can be extended to various material combinations for the fabrication of versatile and functional hybridized nanoarchitecture electrodes^48^,^49^,^50^,^51^.

## Methods

### Materials

Graphite flakes, pyrrole (≥98%), PVA (MW: 89 000–98 000), potassium persulfate (≥99%), phosphorus pentoxide (≥98%), and *N*-methyl-2-pyrrolidone (NMP; 99.5%) were purchased from Sigma-Aldrich. Low–molecular weight PVA (MW: 20 000–30 000) was obtained from Acros, and ferric chloride (97%) and sulfuric acid (95–98%) were obtained from Merck. Poly(vinylidene fluoride) (PVDF; KF1300 binder) was used as a binder. Cellulose paper (pore size: 0.45 μm) and polypropylene (pore size: 8.0 μm) as separators were obtained from Whatman.

### PPy nanosphere synthesis

PPy nanospheres with different diameters were obtained through the following procedure: First, PVA (MW: 20 000–30 000) was dissolved completely in distilled water. To obtain PPy nanospheres with diameters of ~60 and ~100 nm, the PVA was dissolved in distilled water at 3.0:1 and 1.5:1 PVA/water weight ratios, respectively. Ferric chloride was then added to the solutions, followed by pyrrole, at a 3.8:1 ferric chloride/pyrrole molar ratio in which the pyrrole/distilled water volume ratio was 6.3 × 10^−3^:1. Chemical-oxidation polymerization was conducted at 10 °C for 2 h. The resultant PPy nanospheres were washed with excess distilled water and dried in a vacuum oven at room temperature.

### GPPy fabrication

First, graphite was preliminarily treated with small molecule intercalants using the following procedure: Two reagents, i.e., potassium persulfate and phosphorous pentoxide, were dissolved at a 4:1 reagent/graphite weight ratio in a sulfuric acid solution (12.5 mL). Graphite flakes (0.5 g) were added to the solution, which was then stirred for 1 h. The graphite flakes were retrieved via filtering and finally heated at 800 °C for 20 min under a nitrogen atmosphere. To fabricate the GPPy samples, the as-obtained graphite (0.01 g) was mixed with PPy nanospheres in 40 mL of distilled water, and the PPy/graphite weight ratios were controlled to 2:1, 4:1, 6:1, 8:1, and 10:1. The solutions were subjected to mild sonication (frequency: 40 kHz, power: 700 W, benchtop sonicator, Powersonic 420, Hwashin Technology Co., Korea) for 6 h. The resultant products were harvested via filtration and then vacuum dried. The final products were labeled *m*GPPy*n*, where *m* and *n* indicate the PPy/graphite weight ratio and PPy nanosphere diameter (units: nm), respectively.

### Electrochemical measurements

The nanohybrids were dispersed in NMP with PVDF binder (5 wt%) and then coated onto stainless steel to form electrodes. CV and galvanostatic charge/discharge experiments were performed in a three-electrode cell containing 1 M sulfuric acid solution as an electrolyte, and using a Pt counter-electrode and Ag/AgCl reference electrode. A total of six two-electrode cells were built with several different electrolyte, separator, and current collector combinations (Table 1). In four of these cells, either cellulose or polypropylene was used as a separator for the 1 M H_2_SO_4_ or 1 M Na_2_SO_4_ electrolyte with a stainless-steel current collector (thickness: 0.254 mm). In the remaining two cells, a nickel current collector was used with a cellulose or polypropylene separator in 6 M KOH electrolyte.

### Solid-state flexible capacitor cells

8GPPy100 was coated onto stainless-steel foil (area: 8 mm × 15 mm; thickness: 0.0254 mm) as a current collector. Polymer gels were prepared with 1 g PVA (MW: 89 000–98 000), 10 mL Na_2_SO_4_ or H_2_SO_4_ solution (0.08 M), and 20 mL distilled water and immersed in 1 M H_2_SO_4_ or 1 M Na_2_SO_4_ electrolyte. The polymer gel electrolyte was then inserted as both the separator and electrolyte between the electrodes to produce solid-state flexible capacitor cells.

### Characterization

SEM was conducted using a JEOL JSM-7500F microscope in order to observe the morphologies of the products. Raman spectra were obtained with 532.13 nm excitation using a JASCO NRS-5100 spectrophotometer. All electrochemical measurements were conducted using a Metrohm Auto B.V. PGSTAT101 potentiostat/galvanostat. EIS Nyquist plots were recorded within a frequency range of 100 mHz to 1 MHz.

## Additional Information

**How to cite this article**: Lee, Y. *et al.* Nanoparticle-Mediated Physical Exfoliation of Aqueous-Phase Graphene for Fabrication of Three-Dimensionally Structured Hybrid Electrodes. *Sci. Rep.* **6**, 19761; doi: 10.1038/srep19761 (2016).

## Supplementary Material

Supplementary Information

## Figures and Tables

**Table 1:** Specific capacitance and voltage of 8GPPy60 nanohybrid capacitor cells fabricated with different electrolytes, separators, and current collectors.

Sample	Electrolyte	Separator	Current collector	Capacitance (F g^−1^)	Voltage range (V)
1	1M H_2_SO_4_	Cellulose	Stainless steel	245.9	0−0.8
2	1M H_2_SO_4_	Polypropylene	Stainless steel	227.6	0−0.8
3	1M Na_2_SO_4_	Cellulose	Stainless steel	191.1	0−0.8
4	1M Na_2_SO_4_	Polypropylene	Stainless steel	Not measured	—
5	6M KOH	Cellulose	Nickel	263.8	0−0.5
6	6M KOH	Polypropylene	Nickel	284.2	0−0.5
