# Supplementary material for: Nanoparticle-Mediated Physical Exfoliation of Aqueous-Phase Graphene for Fabrication of Three-Dimensionally Structured Hybrid Electrodes
Source: Sci Rep. 2016 Jan 27;6:19761. doi: 10.1038/srep19761 (PMC4728443; doi:10.1038/srep19761)
Supplement: Supplementary Information [file srep19761-s1.pdf]

Supplementary Information

Nanoparticle-Mediated Physical Exfoliation of Aqueous-Phase Graphene for Fabrication of Three-Dimensionally Structured Hybrid Electrodes

Younghee Lee,[b] Hojin Choi,[b] Min-Sik Kim,[b] Seonmyeong Noh,[b] Ki-Jin Ahn,[b] Kyungun Im,[b] Oh Seok Kwon,[c,*] and Hyeonseok Yoon[a,b,*]

[a]Alan G. MacDiarmid Energy Research Institute, School of Polymer Science and Engineering, Chonnam National University, 77 Yongbong-ro, Buk-gu, Gwangju 500-757, South Korea.
E-mail: hyoon@chonnam.ac.kr; Tel: +82-62-530-1778; Fax: +82-62-530-1779

[b]Department of Polymer Engineering, Graduate School, Chonnam National University, 77 Yongbong-ro, Buk-gu, Gwangju 500-757, South Korea.

[c]BioNanotechnology Research Center, Korea Research Institute of Bioscience and Biotechnology, 125 Gwahak-ro, Yuseong-gu, Daejon 305-806, South Korea.
E-mail: oskwon7799@gmail.com; Tel: +82-42-879-8462; Fax: +82-42-879-8594

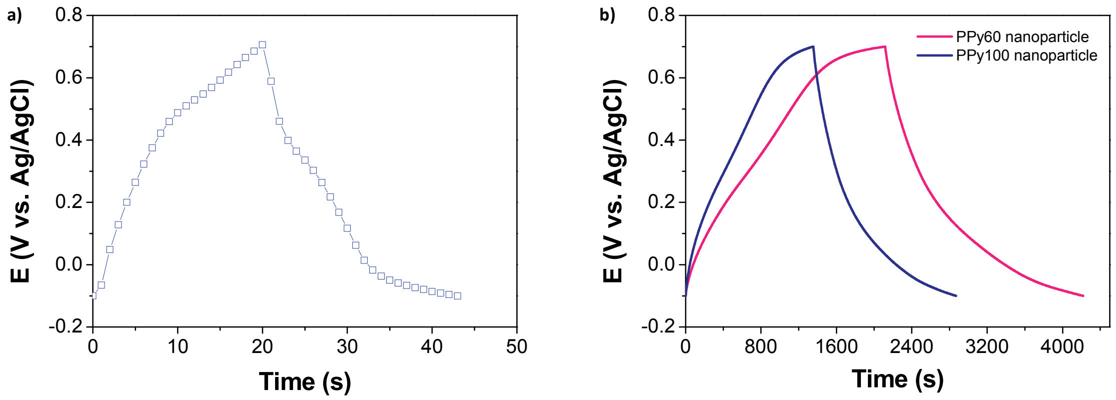

Figure S1. Representative galvanostatic charge/discharge curves of the controls recorded at 0.1 A g^{-1} current density: (a) graphite precursor (exfoliated graphite) and (b) PPy nanospheres.

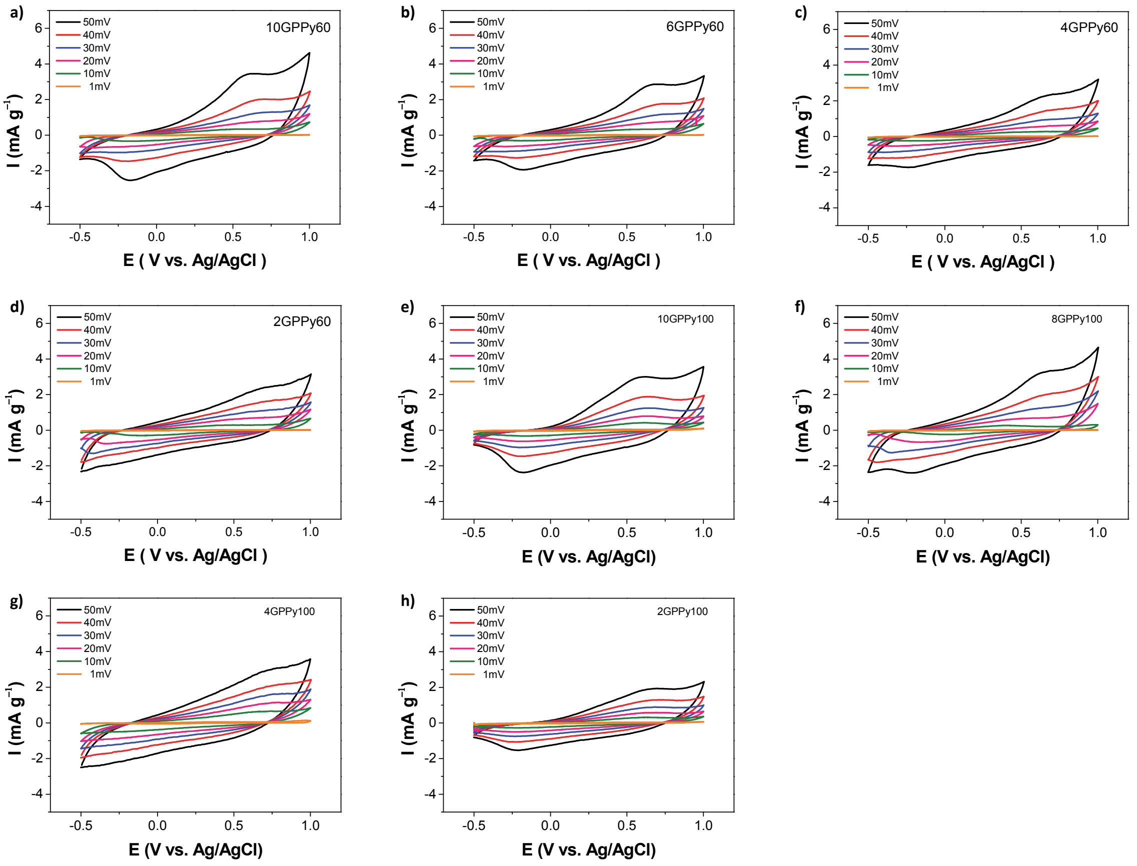

Figure S2. CV curves of (a-d) GPPy60 and (e-h) GPPy100 samples with different PPy-to-graphite weight ratios at scan rates ranging from 1−50 mV s^{-1}: (a) 10:1, (b) 6:1, (c) 4:1 and (d) 2:1; (e) 10:1, (f) 6:1, (g) 4:1 and (h) 2:1.

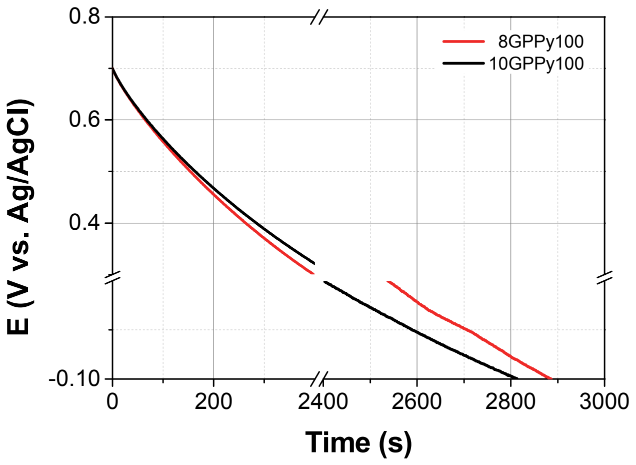

Figure S3. Discharge curves of 8GPPy100 and 10GPPy100.

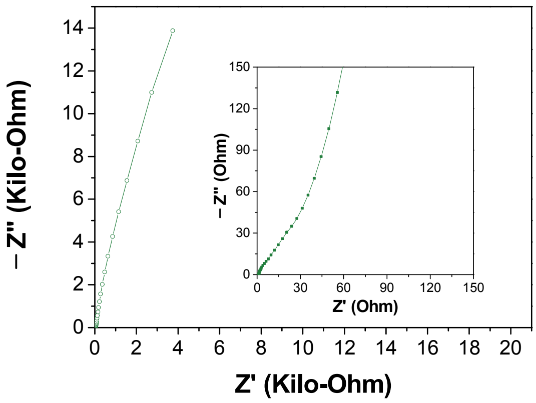

Figure S4. (a) EIS Nyquist plot of pre-exfoliated graphite measured at the same condition as a control.

Table S1. Parameters calculated from EIS measurements using equivalent circuit presented in Figure 6c.

Sample	R_1 (Ω)	R_2 (Ω)	C_1 (μF)	CPE (mMho)
Graphite (pre-exfoliated)	1.15	31.0	5.6	98.7×10^{-3}
2GPPy60	1.18	16.9	11.6	14.1
4GPPy60	1.14	6.9	15.6	17.1
6GPPy60	1.17	5.1	17.9	17.2
8GPPy60	1.18	1.4	20.4	33.5
10GPPy60	1.17	1.7	18.9	31.7
2GPPy100	1.13	22.4	11.5	13.8
4GPPy100	1.19	11.5	12.4	14.3
6GPPy100	1.10	8.5	17.2	16.0
8GPPy100	1.13	2.9	20.1	20.1
10GPPy100	1.19	4.2	17.6	18.0

Table S2. Elemental analysis data of GPPy nanohybrids.*

Sample	Atom weight (wt%)				PPy-to-graphene weight ratio
	N	C	H	S	
2GPPy60	9.917	66.541	4.143	0	2.1
4GPPy60	11.566	60.512	4.471	0	3.8
6GPPy60	12.036	57.868	4.777	0	5.0
8GPPY60	12.242	57.511	4.859	0	5.4
10GPPy60	12.273	57.183	4.774	0	5.5
2GPPy100	10.720	64.388	3.934	0	2.6
4GPPy100	12.104	59.764	4.470	0	4.5
6GPPy100	12.274	58.494	4.705	0	5.1
8GPPY100	12.495	58.317	4.660	0	5.5
10GPPy100	12.519	57.472	4.725	0	5.9

*Thermo Scientific FLASH 2000 CHNS/O analyzer was used.

Assumptions for calculating the packing density of the nanohybrids:

1) At the 10:1 PPy-to-graphite weight ratio, the graphene surface coverage of 60-nm-diameter nanospheres reaches to 100% while that of 100-nm-diameter nanospheres reaches to 90%. The different surface coverage of the two nanospheres is considered in determining the loading amount of the nanosphere. The SEM images of the nanohybrids supports this assumption.

2) The mass density of graphene is 7.6×10^{-8} g cm^{-2}.

3) All nanospheres are well positioned between graphene layers.
